# Preparation and Quality Control of ^68^Ga-Citrate for PET Applications

**Published:** 2015

**Authors:** Ayuob Aghanejad, Amir Reza Jalilian, Khosro Ardaneh, Fatemeh Bolourinovin, Hassan Yousefnia, Ali Bahrami Samani

**Affiliations:** Radiation Application Research School, Nuclear Science and Technology Research Institute, Tehran, Iran

**Keywords:** ^68^Ge/^68^Ga Generator, ^68^Ga-Citrate, Biodistribution, Quality Control

## Abstract

**Objective(s):**

In nuclear medicine studies, gallium-68 (^8^Ga) citrate has been recently known as a suitable infection agent in positron emission tomography (PET). In this study, by applying an in-house produced ^68^Ge/^68^Ga generator, a simple technique for the synthesis and quality control of ^68^Ga-citrate was introduced; followed by preliminary animal studies.

**Methods:**

^68^GaCl_3_ eluted from the generator was studied in terms of quality control factors including radiochemical purity (assessed by HPLC and RTLC), chemical purity (assessed by ICP-EOS), radionuclide purity (evaluated by HPGe), and breakthrough. ^68^Ga-citrate was prepared from eluted ^68^GaCl_3_ and sodium citrate under various reaction conditions. Stability of the complex was evaluated in human serum for 2 h at 370C, followed by biodistribution studies in rats for 120 min.

**Results:**

^68^Ga-citrate was prepared with acceptable radiochemical purity (>97 ITLC and >98% HPLC), specific activity (4-6 GBq/mM), chemical purity (Sn, Fe<0.3 ppm and Zn<0.2 ppm) within 15 min at 500C. The biodistribution of ^68^Ga-citrate was consistent with former reports up to 120 minutes.

**Conclusion:**

This study demonstrated the possible in-house preparation and quality control of ^68^Ga-citrate, using a commercially available ^68^Ge/^68^Ga generator for PET imaging throughout the country.

## Introduction

Considering the intriguing physical properties and availability of gallium-68 as a generator, this isotope has become an interesting nuclide for developing new positron emission tomography (PET) tracers (1). In nuclear medicine, the increasing trend in the production and use of PET radionuclides has provided new opportunities for researchers to focus on the production of new ^68^Ga radiopharmaceuticals, considering the availability and commercialization of ^68^Ge/^68^Ga generators.

^68^Ge decays via pure electron capture (EC) to the ground state of ^68^Ga with a half-life of 270.95 days (2). ^68^Ga in turn decays with a half-life of 67.71 min by a combination of EC and positron emission to the ground state of zinc-68 (^68^Zn). ^68^Ga also decays to an excited state at 1077 keV (with a probability of about 3%) and some higher energy-excited states (with a combined probability of <0.4 %).

For decades, ^67^Ga-citrate has been known as an imaging agent for the detection of infections and inflammations (3). The preparation, quality control, and significance of ^67^Ga-citrate for the evaluation of various infections have been previously investigated (4). After the development of ^68^Ga generators, the application of ^68^Ga-citrate was not promising since most of ^68^Ga images were obtained far beyond the physical half-life of ^68^Ga; as a result, preparation and application of ^68^Ga-citrate were discarded for some time.

However, after the implementation of few clinical trials in various centers, researchers became interested in ^68^Ga infection studies. The preliminary data confirmed the possible role of ^68^Ga-citrate in the diagnosis of bone infections (5). These reports initiated various studies on infectious animals, and different production routes were reported (6-10). Also, some researchers have revealed the application of this tracer for the detection of atherosclerotic plaques in animal models (11).

The area of research is now open to clinical researchers for the evaluation of this tracer in already-confirmed applications of its SPECT homolog (i.e. ^67^Ga-citrate) for conditions such as fever of unknown origin, severe lymphocytic inflammation, autoimmune-based inflammations, chronic pancreatitis, idiopathic pulmonary fibrosis, pulmonary Wegener’s granulomatosis, chronic bronchial asthma, and sarcoidosis (12). On the other hand, the short half-life of ^68^Ga-citrate, which is considered an advantage from a dosimetric point of view, can be a drawback at the same time since it does not allow long uptake duration, which is typical of ^67^Ga-citrate scintigraphy.

**Figure 1:**
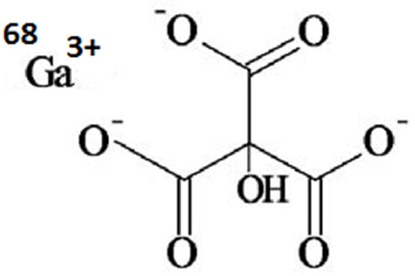
The chemical structure of 68Ga-citrate

In this study, we report the development of an in-house generator, the quality assurance and quality control of eluted ^68^GaCl_3_, and a simple ^68^Ga-citrate production method for any stand-alone PET center, utilizing a ^68^Ga generator. The preclinical evaluation of the ^68^Ga-citrate complex in wild-type rats was also reported (Figure 1).

## Methods

The prototype ^68^Ge/^68^Ga generator (50 mCi/day) was obtained from Pars Isotope Co. Karaj, Iran. Chemicals were purchased from Aldrich chemical Co., Germany. Normal saline, sodium citrate, acetone, and sodium acetate, used for radiolabeling, had high purities and were filtered by 0.22 μm Cativex filters.

Instant thin-layer chromatography (ITLC) was performed by counting Whatman filter papers (No. 2), using a thin-layer chromatography scanner (Bioscan AR2000, Bioscan Europe Ltd., France). Analytical high-performance liquid chromatography (HPLC), used to determine the specific activity, was performed by a Shimadzu LC-10AT pump, equipped with two detector systems, a flow scintillation analyzer (Packard-150 TR), and an ultraviolet–visible spectrophotometer (Shimadzu), using a Whatman Partisphere C-18 column (250×4.6 mm) (Whatman, NJ, USA).

Analytical HPLC was also used to determine the specific radioactivity of the labeled compound. Biodistribution data were acquired by counting normal saline-washed tissues after weighing on a Canberra™ high-purity germanium (HPGe) detector (model GC1020-7500SL). Radionuclidic purity was assessed using the same detector.

For activity measurement of samples, a CRC Capintec Radiometer (NJ, USA) was applied. All calculations and ITLC counting were based on the 511 keV peak. Animal studies were performed in accordance with the Guidelines on the Use of Living Animals in Scientific Investigations (2^nd^ edition) by the United Kingdom Biological Council.

### ^68^Ge/^68^Ga generator

The preparation of the prototype generator has been formerly described in detail; however, few modifications were made to enhance the efficacy of this generator (13). In brief, for a 50 mCi ^68^Ge/^68^Ga generator, metallic gallium powder was melted, transferred to niobium capsules, and then sealed. The target was irradiated at 29 MeV in a 30 MeV IBA cyclotron (effective 22 MeV protons on the target material).

Following irradiation, the target material was dissolved in 12 M H_2_SO_4_, while being gently heated and stirred. For increasing the solubility, 30% hydrogen peroxide was added to the mixture, followed by the addition of 2 M HCl (30 ml). After the completion of dissolution, carbon tetrachloride (150 ml) was added to the mixture. The organic ^68^Ge-containing layer was extracted from ^68^Ga, containing aqueous solution. ^68^Ge was then back-extracted into the aqueous solution, using 0.05 M HCl. The final solution was used for the production of a SnO_2_-based generator.

### Quality control of the product

#### Radionuclide purity

Gamma spectroscopy of the final sample was carried out using an HPGe detector, coupled to a Canberra™ multi-channel analyzer for 1000 sec. Breakthrough was measured by counting the same sample 48 hours after the first test for the detection of small amounts of ^68^Ge in the sample.

#### Chemical purity

This step was carried out to ensure that the values of Sn, Zn, Fe, Ge, and Ga ions, resulting from the target material and presenting in the final product, were within the international limits. Chemical purity was assessed using Inductively-Coupled Plasma Optical Emission Spectrometry (ICP-OES) method. The detection limit of our system was 0.1 ppm for all cations.

### Preparation of ^68^Ga-citrate

The acidic solution of ^68^GaCl_3_ (3-5 mCi in 150µl), eluted by 0.6 M HCl (metal free and ultrapure), was transferred to a 5 ml borosilicate vial and heated to dryness, using a flow of N_2_ gas at 50-60 °C, followed by the addition of sodium citrate solution (300 μL, 0.1 M); the mixture was vortexed at 50 °C for 10-15 min and controlled for radiochemical purity. The active solution with acceptable radiochemical purity was sterile filtered, using a 0.22 micron membrane; also, pH was adjusted to 5.5-7.

### Quality control of ^68^Ga-citrate

#### Radio thin-layer chromatography

A 5 μl sample of the final fraction was spotted on Whatman No. 2 paper and/or silica gel-coated plates, using various mobile phases.

#### HPLC

HPLC was performed with a flow rate of 1 ml/min under 130 kgF/cm^2^ pressure for 20 min. HPLC was performed on the final preparation, using a mixture of water and acetonitrile with a ratio of 3:2 (v/v) as the eluent, using reversed-phase Whatman Partisphere C_18_ column (4.6×250 mm).

### Stability tests

The stability of the complex was assessed using conventional ITLC method (14). A sample of ^68^Ga-citrate (37 MBq) was kept at room temperature for up to 2 hours, while being checked by ITLC at different time intervals in order to check the stability of the final product, using the above-mentioned chromatography system.

#### In vitro stability of ^68^Ga-citrate in the presence of human serum

The final solution (200 µCi in 50 µL) was incubated in the presence of freshly prepared human serum (300 µL) (purchased from Iranian Blood Transfusion Organization, Tehran) and kept at 37 °C for 2 hrs. Every 30 min, trichloroacetic acid (10%, 100 µL) was added to a portion of the mixture (50 μl) and the mixture was centrifuged at 3000 rpm for 5 min, followed by decanting the supernatant from the debris. Stability was determined by performing frequent ITLC analysis of supernatant, using the ITLC system.

### Biodistribution in wild-type rats

The distribution of the radiolabelled complex, as well as free ^68^Ga cation, was determined in rat tissues. The total amount of radioactivity injected into each rat was measured by evaluating the 1 ml syringe before and after the injection in a dose calibrator with fixed geometry. The animals were sacrificed by CO_2_ asphyxiation at selected time intervals after injection (n=3 for each time interval). The tissues (blood, heart, lung, brain, intestine, feces, skin, stomach, kidneys, liver, muscle and bone) were weighed and rinsed with normal saline and their specific activities were determined as the percentage of injected dose per gram of tissues, using an HPGe detector, equipped with a sample-holder device.

## Results

### 

#### Quality control of ^68^Ga generator

**Figure 2:**
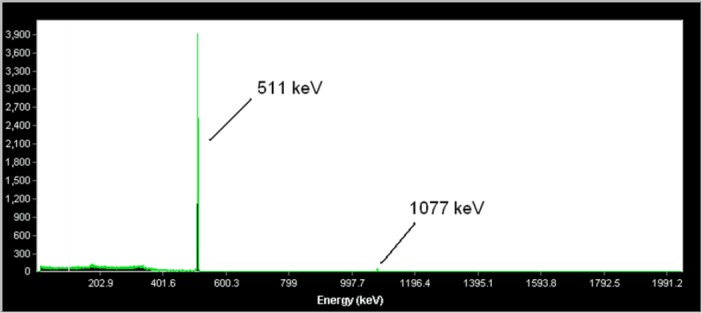
Gamma spectrum for ^68^GaCl_3_ solution, eluted from the generator, used in radiolabeling

Radiochemical separation of ^68^Ge from irradiated natural Ga was performed via a two-step extraction method in an organic solution, followed by back extraction with a 96% yield. The presence or absence of ^68^Ga and ^68^Ge was assessed at each step, using the HPGe detector (Figure 2).

Radionuclidic control indicated the presence of 511 and 1077 keV energies, which originated from ^68^Ga and showed a radionuclidic purity higher than 99% (E.O.S.). For the quality control of the ^68^GaCl_3_ solution, a time-activity study was performed on the eluted sample after more than 10 half-lives of ^68^Ga in order to assess the ^68^Ge breakthrough. The data were recorded up to 8 days after elution. Calculations showed that the ^68^Ge/^68^Ga activity ratio was 1.6600×10^-5^ at the time of elution.

The concentrations of tin (from the generator material), iron (from the sealing parts and acid impurities), zinc (as a decay product), and gallium (as the target material) were determined, using the ICP-OES method (Table 1).

**Table 1:** ICP-OES mass data on various elutions of prototype generator in our study

Element	Generator 1 (mg/L)	Generator 2 (mg/L)	Generator 3 (mg/L)
**Fe**	0.557	0.432	0.230
**Sn**	0.340	<0.1	<0.1
**Zn**	0.284	0.110	<0.1
**Ga**	<0.1	<0.1	<0.1
**Ge**	<0.1	<0.1	<0.1

**Figure 3:**
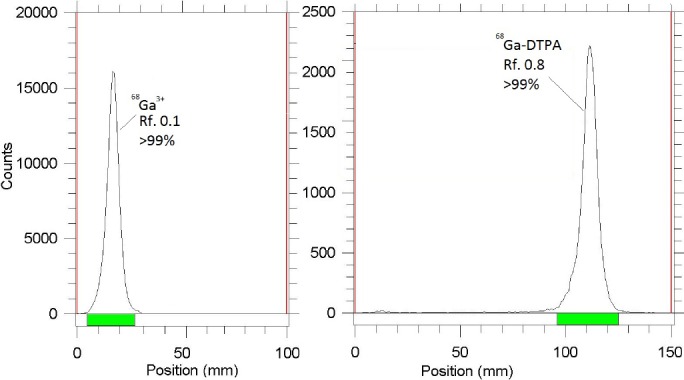
ITLC chromatograms of ^68^GaCl_3_ solution in 10% ammonium acetate: methanol (1:1) (left) on Silica gel sheets and in 10 mM DTPA solution (pH~4) on Whatman No. 1 paper (right)

The radiochemical purity of ^68^GaCl_3_ solution was evaluated in two solvents. In 10 mmol*L^-1^ diethylenetriaminepentaacetic acid (DTPA) aqueous solution (solvent 1), free ^68^Ga^3+^ was coordinated to more lipophilic moiety as ^68^Ga (DTPA)^2-^ and migrated to a higher R_f_ value (Figure 3). The small radioactive fraction remaining at the origin could be associated with colloids, since in the presence of a strong complexing agent (i.e., DTPA), existence of other ionic species other than ^68^Ga (DTPA)^2-^ is rare. On the other hand, a 1:1 ratio of 10% ammonium acetate to methanol mixture (solvent 2) was used for the determination of radiochemical purity.

The fast-eluting species were possibly ^68^Ga and other ionic forms of ^68^Ga including ^68^GaCl_4_^-^ (if existed), which remained at the origin (R_f_= 0), and colloids (not detected) (Figure 3). The differences in the impurity peaks in the two chromatograms could be to some extent related to the presence of colloidal impurity, which was insignificant. Additionally, the insignificant amount of activity (about <1%) could be attributed to other ionic impurities.

### Radiolabeling

Considering the limited half-life of ^68^Ga, in order to obtain the best reaction yields in the shortest possible time, optimization studies were conducted, using various citrate sources (i.e., sodium citrate and/or citric acid) and citrate anion concentrations at different temperatures. In order to select the best solid phase/eluent for fast and efficient radiochemical purity control, various solvent mixtures and stationary phases (silica gel and Whatman paper) were employed.

**Figure 4:**
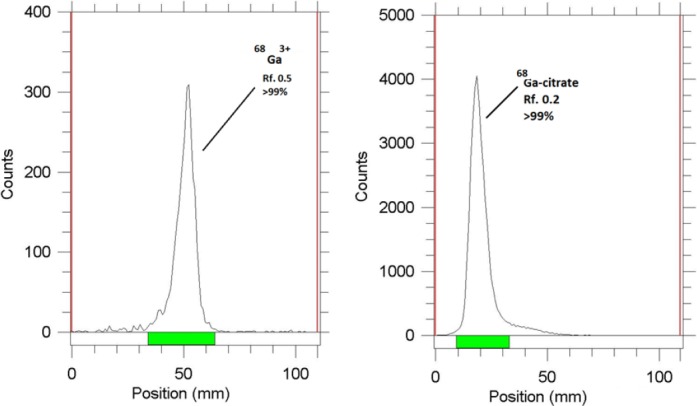
ITLC chromatograms of ^68^GaCl_3_ (left) and 68Ga-citrate (right) solutions in acetone/glacial acetic acid (3:1) on Whatman paper

Table 2: demonstrates the R_f_ for various solvent mixtures, as well as the stationary phases for radiolabeling reactions. However, most of the systems did not provide reproducible and/or clear results for Ga cation and the labeled compound. The best system was considered a mixture of acetone and glacial acetic acid (3:1) in both silica gel and Whatman paper in the stationary phase. Finally, due to better peak distinction and rapid elution time, Whatman paper was selected (Figure 4).

**Table 2:** Chromatographic properties of systems used for the determination of ^68^Ga-citrate radiochemical purity

Mobile phase	Stationary phase	^68^Ga-citrate (R_f_)	^68^Ga-chloride (R_f_)
Methanol/ammonium acetate 10% (1:1)	Whatman	0.45	0.70
Sodium citrate (0.1 M)	Whatman	0.63	0.70
DTPA (1 mM) pH=5	Whatman	0.75	0.80
DTPA (10 mM) pH=5	Whatman	0.80	0.85
Methanol/glacial acetic acid (9:1)	Whatman	0.26	0.50
Sodium acetate (1.5 g)+ acetic acid (0.58 ml) in 100 ml of water	Silica gel	0.40	0.50
Sodium acetate (1.5 g)+ acetic acid (0.58 ml) in 100ml of water	Whatman	0.70	0.40
Acetone/glacial acetic acid (3:1)	Whatman	0.10	0.45
Acetone/glacial acetic acid (3:1)	Silica gel	0.15	0.07
Sodium acetate (0.16 M)	Whatman	0.80	0.53
Sodium acetate (0.16 M)	Silica gel	0.15	0.07

**Figure 5:**
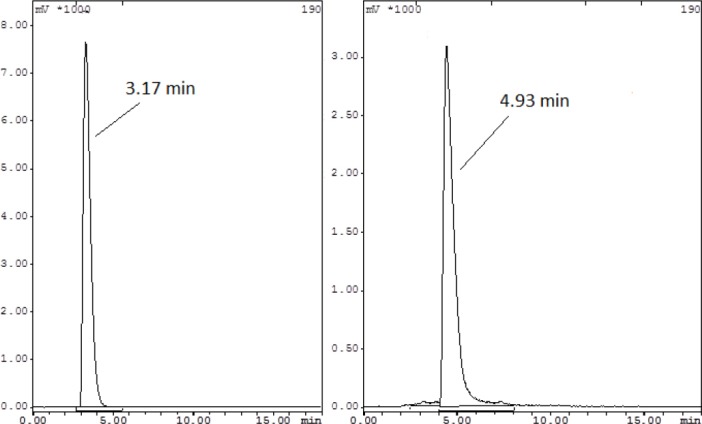
HPLC chromatogram of ^68^Ga chloride (left) and ^68^Ga-citrate (right) solutions on a reversed-phase column, using acetonitrile+0.1% trifluoroacetic acid/water+0.1% trifluoroacetic acid (90:10)

Although the ITLC studies approved the production of the radiolabeled compound, HPLC studies demonstrated the existence of radiolabeled species, using a scintillation detector. A more fast-eluting compound at 3.17 min (using the scintillation detector) was observed for free gallium-68 cation, while for ^68^Ga-Citrate, a second peak was eluted at minute 4.93, using the scintillation detector (Figure 5).

Both species were ionic and possibly, using an ion chromatographic column would be of great value for characterization. However, in our setup, at 5-6 min elution time, the two peaks were distinguishable, using the mentioned system.

### Biodistribution

**Figure 6:**
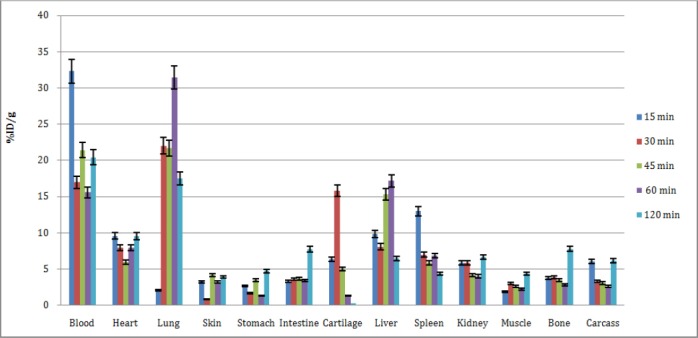
The ID/g percentages of ^68^Ga-citrate in the tissues of mice at 15-120 min after the injection

Biodistribution study was performed for ^68^Ga-citrate. The percentages of injected dose per gram (%ID/g) are summarized in Figure 6. As previously reported, ^68^Ga was excreted majorly from the gastrointestinal tract with high blood content due to transferrin binding at early time intervals (3.2% ID/g at minute 15). Moreover, significant lung, bone, and stomach activities were observed; however, kidney was not a significant accumulation site.

Liver was a major accumulation site for transferrin and many radiolabeled proteins. With respect to the ferric ion-mimicking of Ga cation in the body, the liver could be a major accumulation site within 60 min (17% ID/g). Lung and spleen were two important reticuloendothelial organs in which macrophage cells were located. The mechanism of Ga accumulation in WBCs and macrophage cells has been well documented (15), indicating high lung uptake (33% ID/g at 15 min). Significant cardiac uptake was also attributed to ferric cation homology (10% ID/g at 15 and 120 minutes).

## Discussion

The results showed better chemical purities for all cation impurities during the development of generators with different particle sizes and packing procedures. As a radiolabeling source for ^68^Ga-based radiopharmaceuticals, especially ligands with very low molarities (including peptides), this chemical purity is crucial and sufficient for the procedure.

The United States Pharmacopeia has not yet approved the ^68^Ga generator; however, the European Pharmacopoeia published a monograph on ^68^Ga edotreotide injection in 2011, covering all aspects of this radiopharmaceutical without pointing out metal impurities (16). On the other hand, other available publications on generator production only focus on the breakthrough of ^68^Ge and its half-life. In recent works, the best chemical yields have been reported to be less than 0.1 ppm. So far, no pharmacopeial limit for ^68^Ga generators has been reported in pharmacopoeial references.

One important issue in the elution process was determining the radiochemical form of ^68^Ga. In highly acidic elutions, the formation of other ionic complexes from the generator including ^68^GaCl_4_^-^ was possible. In many cases, this species, present in the solution, did not participate in the complexation process. Dilution and/or pH changes were also not possible due to the formation of volatile species and/or high ionic strength of the solution, which is not recommended for radiopharmaceuticals.

As it was shown, although the reaction would take place in the presence of various citrate sources, the best yields were obtained by using sodium citrate. On the other hand, the reaction was completed within an hour at room temperature; however, applying gentle heat in a water bath at 50 °C provided acceptable radiochemical yields within 10-15 min.

For the best performance, the generator must be eluted daily (even if not used every day) in order to remove the unwanted decay or radiation-induced impurities. Also, when starting the preparation procedure, the first 1-2 ml fractions can be discarded. At a typical run, the 3-5 fractions show the best specific activities daily available.

Rizzello et al. reported a radiochemical yield of 64% for ^68^Ga-citrate production with an average activity of about 970 MBq available and 98% radiochemical purity (8). On the other hand, our data demonstrated a significant enhanced radiochemical yield of 72% with almost the same radiochemical purity (>98% HPLC) and high specific activity (4-6 GBq/mM).

In many reports, the biodistribtion of free gallium cation has been reported in a 72 h time span, using ^67^Ga (17). ^67^Ga is excreted majorly from the gastrointestinal tract; thus, colon and stool uptakes are significant, while blood stream activity is not major in the urinary system. However, for Ga-citrate, data are acquired in a shorter time span. Since Ga as a homolog of ferric cation is bound to plasma proteins, the blood content is high up to 2 hours. Also, the fate of transferrin and similar proteins is determined in the liver, which is the final accumulation site as already seen in case of ^68^Ga.

## Conclusion

In this study, ^68^Ga-citrate was prepared with acceptable radiochemical purity (>97 ITLC and >98% HPLC), specific activity (4-6 GBq/mM), and chemical purity (Sn, Fe <0.3 ppm and Zn<0.2 ppm) within 15 min at 50 °C. The biodistribution of ^68^Ga-citrate was consistent with former reports up to 120 min. This study demonstrated possible in-house preparation and quality control of ^68^Ga-citrate, using commercially available ^68^Ge/^68^Ga generator for PET imaging throughout the country.
